# Effects of isoflavone and probiotic intake on calcium transport and bone metabolism biomarkers in female rats

**DOI:** 10.1002/fsn3.3571

**Published:** 2023-07-23

**Authors:** Iskandar Azmy Harahap, Maciej Kuligowski, Marcin Schmidt, Paweł A. Kołodziejski, Joanna Suliburska

**Affiliations:** ^1^ Department of Human Nutrition and Dietetics, Faculty of Food Science and Nutrition Poznan University of Life Sciences Poznan Poland; ^2^ Department of Food Technology of Plant Origin, Faculty of Food Science and Nutrition Poznan University of Life Sciences Poznan Poland; ^3^ Department of Biotechnology and Food Microbiology, Faculty of Food Science and Nutrition Poznan University of Life Sciences Poznan Poland; ^4^ Department of Animal Physiology, Biochemistry and Biostructure, Faculty of Veterinary Medicine and Animal Science Poznań University of Life Sciences Poznan Poland

**Keywords:** bone metabolism biomarkers, calcium transports, isoflavones, *Lactobacillus acidophilus*, rats

## Abstract

Calcium is essential for maintaining bone health as it contributes to bone formation, remodeling, strength, and density. This study investigated the effect of isoflavones and probiotics on calcium transporters' gene expression, serum calcium levels, and bone metabolism biomarkers in healthy female rats. Forty‐eight female Wistar rats were classified into six groups. Bone metabolism biomarkers (pyridinoline, deoxypyridinoline, parathyroid hormone, and osteocalcin) and serum calcium levels were measured by enzyme‐linked immunosorbent assay (ELISA) and atomic absorption spectroscopy (AAS), respectively. Gene expression of calcium transporters (Trpv5 and Trpv6) was evaluated in duodenum and jejunum tissue samples using quantitative polymerase chain reaction (qPCR). Trpv5 and Trpv6, epithelial calcium channels, play a crucial role in calcium transport and homeostasis in the body. The study consisted of a1‐week adaptation period for the rats to adjust to the controlled conditions, followed by an 8‐week intervention phase. The daidzein and genistein group showed a significant increase in the gene expression of the Trpv6 transporter in the duodenum and a marked decrease in serum pyridinoline levels compared to the control group. The tempeh and soybean groups showed a significant decrease in the gene expression of the Trpv5 calcium transporter in the jejunum. However, no significant influence of the *Lactobacillus acidophilus* diet on calcium transport and bone metabolism biomarkers was observed in the *L. acidophilus* group. The correlation analysis showed a significant positive relationship between serum calcium, bone metabolism biomarkers, and calcium transporters. In conclusion, our study demonstrates that the daidzein and genistein diet improves calcium transport in the duodenum and reduces pyridinoline serum concentrations, while tempeh and soybean diets reduce calcium transport in the jejunum. However, the combination of daidzein, genistein, and *L. acidophilus* did not demonstrate a synergistic effect on calcium transport and bone metabolism, suggesting that further investigations are needed to elucidate their potential interactions.

## Graphical abstract

This study investigated the effects of isoflavones and probiotics on calcium transport and bone metabolism in female rats. The results showed that daidzein and genistein improved calcium transport in the duodenum and reduced pyridinoline serum concentrations, while tempeh and soybean diets reduced calcium transport in the jejunum. However, no synergistic effect of the combination of daidzein, genistein, and *L. acidophilus* on calcium transport and bone metabolism was observed.

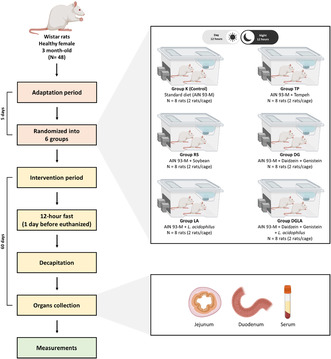

## 1 INTRODUCTION

Calcium deficiency or insufficient intake contributes to osteoporosis and an increased risk of fractures (Aaseth et al., 2012). Postmenopausal women face a significantly higher risk of developing osteoporosis and fractures due to decreased estrogen levels, impaired bone remodeling, reduced calcium absorption, and other contributing factors (de Villiers & Goldstein, 2021). Calcium deficiency poses a heightened risk in the elderly due to physiological and metabolic changes, as well as increased calcium requirements associated with aging. These changes can lead to inadequate calcium intake, impaired calcium absorption, and altered calcium metabolism, contributing to the risk of deficiency. Calcium supply and its bioavailability significantly affect the appropriate level of calcium and bone health (Sozen et al., 2017). Isoflavones and probiotics have been suggested to play a significant role in the prevention of osteoporosis by influencing calcium absorption and bone metabolism. Isoflavones, such as genistein and daidzein, have been shown to mimic the effects of estrogen in the body, promoting osteoblast activity and reducing bone resorption. They may also enhance intestinal calcium absorption and inhibit bone loss. Probiotics, such as *Lactobacillus acidophilus*, can enhance calcium solubilization, modulate gut microbiota, and improve intestinal health, thereby potentially enhancing calcium absorption and bone mineral density (Harahap & Suliburska, 2021).

The existing recommendations for the treatment and prevention of postmenopausal osteoporosis include estrogen treatment and pharmacological medications. Although estrogen treatment can help preserve or improve bone mineral density (BMD), it also increases the risk of reproductive cancer (Shah & Wong, 2006). Furthermore, though medications such as bisphosphonates, calcitonin, and denosumab can be prescribed for postmenopausal osteoporosis, their long‐term usage can lead to side effects, infusion frequency requirements, and other issues (Cosman et al., 2014). The current recommendations do not sufficiently address the prevention of osteoporosis hazards (Ebeling et al., 2022). An appropriate ratio of bone resorption by osteoclasts to bone synthesis by osteoblasts needs to be maintained to achieve strong bones. Hormones, immunological cells, and the digestive system play roles in the regulation of bone resorption and synthesis. Bone cells receive signals to commence mineralization from endocrine hormones (such as incretins and serotonin) produced in the intestine (McCabe & Parameswaran, 2018). Therefore, there is a significant clinical need for developing effective medicines with fewer side effects that are suitable for long‐term usage to preserve bone health during and after menopause.

Bone is a mineralized tissue that supports and feeds the skeleton. It constantly remodels the subtle balance between osteoblasts and osteoclasts. Parathyroid hormone (PTH) is a hormone that regulates plasma calcium levels by facilitating bone resorption and renal calcitriol synthesis (de Brito Galvao et al., 2013), whereas osteocalcin (OC) is an osteoblast‐specific matrix protein (Qin et al., 2004). Bone metabolism biomarkers are diagnostic tools that maintain the general well‐being of individuals. Deoxypyridinoline (DPD) and pyridinoline (PYD) are cross‐linking molecules found in collagen fibers and play a critical role in maintaining the integrity and strength of the extracellular matrix in bone tissue. DPD is synthesized from side chains of two hydroxylysine molecules and one lysine molecule, whereas PYD is formed from three hydroxylysine molecules. Due to their high remodeling rates, bones are widely recognized as one of the most crucial sources (Shankar Ram et al., 2015).

Trpv5 and Trpv6 are highly selective for calcium ions and respond strongly to 1,25‐dihydroxy vitamin D_3_ at the apical membrane of calcium‐ion‐transporting epithelia. They are effective calcium ion entry channels in the early stages of transcellular calcium transport routes in intestinal absorption, renal calcium reabsorption, and placental calcium transfer to the fetus, among others (Peng, 2011). The transcellular component of calcium absorption decreases along the small intestine, with approximately 50% occurring in the duodenum, 20% in the jejunum, and no significant contribution in the ileum (Kellett, 2011).

Therapeutic nutrients such as probiotics and isoflavones may be useful in controlling calcium absorption and bone metabolism. Probiotics can regulate calcium uptake in paracellular and transcellular pathways. Isoflavones facilitate calcium homeostasis by mobilizing calcium from skeletal muscles. *Lactobacillus* and *Bifidobacteria* promote the enrichment and diversity of gut microbiota, leading to enhanced immune function and improved bone health (Harahap & Suliburska, 2023). They contribute to the proliferation of beneficial bacteria, such as Bacteroidetes and Firmicutes, while inhibiting the growth of pathogenic bacteria. Moreover, isoflavones and their metabolites have been shown to enhance bone mineral density by stimulating osteoblast activity, inhibiting bone resorption, and regulating bone remodeling processes (Harahap & Suliburska, 2021).

Furthermore, probiotics may reduce bone loss after menopause by increasing the expression of tight junction proteins in the gut and reducing antigen transport to the intestines, and activating immune cells in them (Amin et al., 2020). Osteoprotective characteristics of *Lactobacillus acidophilus* (LA) have been shown to benefit bone health. Ovariectomized mice that received LA showed improved bone microarchitecture, mineral density, and heterogeneity (Dar et al., 2018). Daidzein is present in a variety of soy products, including fermented soy, especially tempeh (TP), in which the level of isoflavones is significantly higher than in unfermented soy (Kuligowski et al., 2017). Gut bacteria ferment daidzein into equol (Harahap & Suliburska, 2022). Equol protects ovariectomized mice from developing brittle bones (Fujioka et al., 2004).

Numerous previous studies have investigated calcium absorption in healthy female rats (Cashman & Flynn, 1996; Shah et al., 1990; Wood et al., 1998). Health‐promoting effects of the intake of isoflavones and probiotics on calcium status is an emerging research topic. This study investigates the effect of isoflavones and probiotics on calcium transport and bone metabolism in healthy female rats, which is an emerging research topic. While previous studies have explored calcium absorption in healthy female rats, the health‐promoting effects of isoflavones and probiotics on calcium status are relatively new areas of investigation. The findings of this study will contribute to understanding the potential benefits of isoflavones and probiotics on the organism, informing further research directions in this field.

## 2 MATERIALS AND METHODS

This section provides the overall approach and methodology employed in the study. It details the use of chemicals, outlines the experimental design, specifies the duration of the study, and highlights any interventions or treatments administered. Moreover, this section serves as a foundation for understanding the subsequent detailed analyses and measurements.

### 2.1 Materials

AIN 93M (Zoolab), soybean (*Glycine max*) of the Augusta variety (Department of Genetics and Plant Breeding, Poznań University of Life Sciences, Poland), daidzein, and genistein (DG; LC Laboratories) were used as ingredients of the diet for rats. Meanwhile, tempeh (fermented soybean with *Rhizopus oligosporus* NRRL 2710) and probiotics (*Lactobacillus acidophilus* DSM20079) were prepared in accordance with our previously conducted experiments (Harahap, Kuligowski, Schmidt, Kurzawa, et al., 2023; Harahap, Kuligowski, Schmidt, & Suliburska, 2023).

In the tempeh preparation process, soybeans were dehulled, boiled, and cooled. After inoculating the soybeans with *R. oligosporus* NRRL 2710, the mixture was placed in disposable Petri dishes. Fermentation occurred at 30 ± 1°C for 24 ± 1 h. Finally, the tempeh samples were frozen, freeze‐dried, and powdered (Harahap, Kuligowski, Schmidt, Kurzawa, et al., 2023; Harahap, Kuligowski, Schmidt, & Suliburska, 2023).

*Lactobacillu acidophilus* DSM20079 was prepared by inoculating a freeze‐dried stock in MRS broth and incubating for 1 h at room temperature. The suspension was spread on MRS agar and incubated at 37°C for 24 h. A single colony was inoculated in 10 mL of MRS broth and incubated at 37°C for 18 h. Cells were harvested, washed, and resuspended in a mix containing skim milk powder and maltodextrin. The mixture was freeze‐dried, and viable counts were determined. The final probiotic preparation contained corn starch and had a concentration of 10^10^ CFU/g (Harahap, Kuligowski, Schmidt, Kurzawa, et al., 2023; Harahap, Kuligowski, Schmidt, & Suliburska, 2023).

### 2.2 Preparing the study, conditioning the environment, and adapting the rats

The local ethical committee in Poznan, Poland agreed on conducting this study. This study was conducted in accordance with the National Institutes of Health's Guide for the Care and Use of Laboratory Animals (NIH Publications No. 80‐23, Revised 1978), the Directive 2010/63/EU of the European Parliament, the Council of 22, September 2010 on the Protection of Animals Used for Scientific Purposes, and Polish law. Animal experiments adhered to the Animal Research: Reporting of In Vivo Experiments (ARRIVE) guidelines.

**FIGURE 1:**
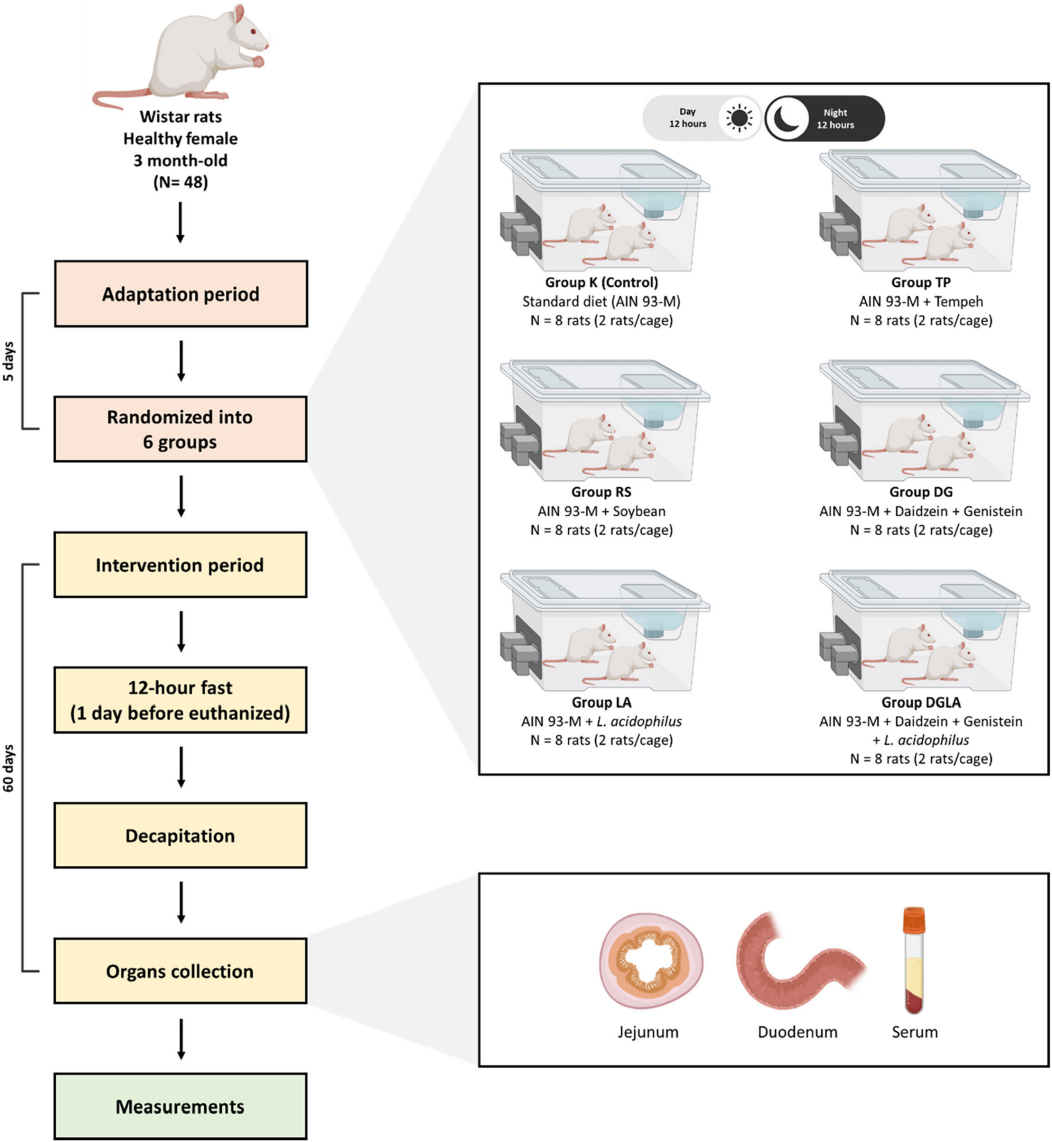
Flow diagram of diet intervention in rats.

The animal laboratory at the Department of Human Nutrition and Dietetics of Poznan University of Life Sciences provided a secure and stable setting for the rats. Throughout the adaption period and the experiment, the rats were maintained in a room with a constant temperature of 21 ± 2°C, relative humidity of 55–65%, and a light/dark cycle of 12 h. During both adaptation and intervention phases, the rats were housed in pairs in enameled stainless‐steel cages to prevent the ingestion of metal particles. A total of 48 three‐month‐old female Wistar rats were used in this study. During the first week in the laboratory, the rats adapted to the controlled conditions. A flowchart depicting the research process is shown in Figure 1.

### 2.3 Preparing the diets and grouping the rats

After the adaptation period, all rats were weighed on a calibrated scale and classified into six groups (eight rats each) based on their body weight. The standard diet (AIN 93M) was provided to the control group (K). The TP group was given AIN 93M with tempeh flour (250 g/kg of standard diet). The RS group received AIN 93M with soybean (250 g/kg of standard diet). To incorporate an inclusion level of 250 g/kg of soybean or tempeh flour in the AIN93M diets, we carefully formulated the diets by adding 250 g of the respective ingredient per kilogram of the AIN93M diet replacing starch. This approach ensured that the diets contained the desired amount of soybean or tempeh flour while maintaining consistency in the overall nutritional composition. The DG group was fed AIN 93M with daidzein (10 mg/kg of standard diet) and genistein (100 mg/kg of standard diet). The LA group was fed AIN 93M with *L. acidophilus* (10^10^ CFU/day), and the DGLA group received AIN 93M with the combination of daidzein (10 mg/kg of standard diet), genistein (100 mg/kg of standard diet), and *L. acidophilus* (10^10^ CFU/day). Comparable components were present in the K, DG, LA, and DGLA diets. TP contained significantly higher concentrations of both daidzein and genistein than RS. The nutritional components and calcium contents are shown in Table 1 from our previous study (Harahap, Kuligowski, et al., 2022; Harahap, Landrier, & Suliburska, 2022).

**TABLE 1:** Nutritional compositions (Harahap, Kuligowski, et al., 2022; Harahap, Landrier, & Suliburska, 2022 with modifications).

Parameters	Unit	K	TP	RS	DG	LA	DGLA
Protein	(g/100 g)	14.95 ± 0.64^a^	23.56 ± 0.83^c^	21.04 ± 0.89^b^	14.11 ± 0.14^a^	14.19 ± 0.17^a^	14.16 ± 0.34^a^
Fat	(g/100 g)	4.28 ± 0.19^a^	7.61 ± 0.23^c^	5.14 ± 0.02^b^	4.51 ± 0.05^a^	4.46 ± 0.17^a^	4.61 ± 0.08^a^
Insoluble fiber	(g/100 g)	5.92 ± 0.45^a^	11.61 ± 0.75^b^	14.88 ± 0.66^c^	5.67 ± 0.01^a^	5.71 ± 0.02^a^	5.98 ± 0.13^a^
Soluble fiber	(g/100 g)	1.87 ± 0.03^a^	3.78 ± 0.04^c^	2.76 ± 0.31^b^	1.92 ± 0.05^a^	1.83 ± 0.03^a^	1.86 ± 0.08^a^
Fiber	(g/100 g)	7.78 ± 0.42^a^	15.38 ± 0.78^b^	17.64 ± 0.96^c^	7.58 ± 0.04^a^	7.54 ± 0.01^a^	7.83 ± 0.07^a^
Carbohydrates	(g/100 g)	70.78 ± 0.62^c^	59.37 ± 1.20^a^	63.13 ± 0.97^b^	71.28 ± 0.20^c^	71.34 ± 0.61^c^	71.07 ± 0.32^c^
Ash	(g/100 g)	2.68 ± 0.07^ab^	2.92 ± 0.09^b^	3.26 ± 0.12^c^	2.45 ± 0.08^a^	2.51 ± 0.15^a^	2.47 ± 0.04^a^
Energy	(kcal/100 g)	346.45 ± 1.83^c^	337.89 ± 3.18^b^	311.49 ± 3.37^a^	351.83 ± 0.57^c^	352.14 ± 0.72^c^	351.17 ± 0.68^c^
Calcium	(mg/100 g)	495.21 ± 5.06	493.71 ± 4.28	492.71 ± 6.24	499.83 ± 9.27	491.38 ± 2.18	495.79 ± 0.76

*Note*: Tukey's test for ANOVA was used. ^a–c^ are mean values significantly different for *p* < .05.

*Abbreviations*: DG, daidzein and genistein; DGLA, daidzein, genistein, and *Lactobacillus acidophilus*; K, control; LA, *Lactobacillus acidophilus*; RS, soybean; TP, tempeh.

We selected the dosages of isoflavones and probiotics based on previous studies that reported their beneficial effects on bone health in rodents and humans (Nakajima et al., 2005). Based on the analysis of isoflavones content (daidzein and genistein) in tempeh, the tempeh flour was determined at 250 g (Harahap, Kuligowski, et al., 2022; Harahap, Landrier, & Suliburska, 2022). For probiotics, we referred to a study by Dar et al. (2018) where ovariectomized female mice were fed with diets containing a dosage of *L. acidophilus* (10^9^ CFU/day) for 6 weeks. With a dose of 10^9^ CFU/day, the authors observed a reduction in osteoclastogenic factor expression and an increase in antiosteoclastogenic factor expression. In addition, compared to the low dose (2.5 × 10^9^ CFU/day), the effect of the high dose of probiotics (1 × 10^10^ CFU/day) was more noticeable in postmenopausal women (Szulińska et al., 2018). Therefore, we chose the dosage of *L. acidophilus* 10^10^ CFU/day in our study. Each group was weighed weekly, and their food intake was documented daily. All groups were given free access to the diet and distilled water.

### 2.4 Decapitating the rats

After the abovementioned intervention for 8 weeks, the rats were subjected to fasting for 12 h. A period of fasting allows to obtain reliable results from biochemical blood tests (Nowland et al., 2011). Afterward, their body mass was measured, and then, they were decapitated. A section was carried out immediately following decapitation.

### 2.5 Collecting serum and organs

The serum was collected in sterilized tubes, and the blood was left to clot at room temperature for 30 min. To separate the blood cells, the samples were centrifuged at 4°C for 15 min at 2000 rpm. Then, the supernatants were collected and frozen at −80°C for later study. The duodenum and jejunum were dissected, cleansed in saline, weighed, and frozen at −80°C for later use.

### 2.6 Determination of serum calcium level

The serum calcium level was measured using flame atomic absorption spectrometry (AAS‐3, Carl Zeiss). The assay for the determination of the serum calcium level was calibrated at a wavelength of 422.7 nm. This method's reliability was validated by the 91% accuracy for calcium in the certified reference material Bovine liver 1577C (Sigma‐Aldrich).

### 2.7 Determination of levels of bone metabolism biomarkers in serum

Commercial enzyme‐linked immunosorbent assay kits purchased from Qayee Bio‐Technology Co., Ltd., and absorption spectrophotometry (LEDetect96, Labexim) were used to measure the serum concentrations of PYD, DPD, PTH, and OC.

### 2.8 Determination of calcium transporters

Quantitative real‐time polymerase chain reaction (RT‐PCR) was used to determine the presence of calcium transporters. Total RNA was extracted using EXTRAzol (DNA Gdansk). Duodenum and jejunum tissues were lysed by adding EXTRAzol to separate PCR tubes. Mechanical homogenization of sample material was performed using TissueLyser II (Qiagen). Using a High‐Capacity cDNA Reverse Transcription Kit (Life Technologies), 1 μg of total RNA was reverse‐transcribed into cDNA. QuantStudio 12K Flex™ was used to conduct RT‐PCR of the collected DNA using gene‐specific primers and 5 XHOT FIREPol® Eva‐Green®qPCR Mix Plus (ROX). Melting points (transition rate of 0.1 C/s) of the DNA were measured to examine the specificity. The delta–delta CT method was used to analyze the relative gene expression using Gapdh as a standard. The complete list of PCR primers is presented in Table 2. Trpv5 and Trpv6 mRNA levels were expressed as arbitrary units relative to Gapdh mRNA levels.

**TABLE 2:** Primer sequences.

Target	Forward primer	Reverse primer
Gapdh	TGACTTCAACAGCGACACCCA	CACCCTGTTGCTGTAGCCAAA
Trpv5	CGAGGATTCCAGATGC	GACCATAGCCATTAGCC
Trpv6	GCACCTTCGAGCTGTTCC	CAGTGAGTGTCGCCCATC

### 2.9 Statistical analysis

The Shapiro–Wilk method was used to evaluate the normal distribution of the variables. Statistically significant differences were determined using an analysis of variance along with Tukey's post hoc test for differences. All differences were considered statistically significant at the 5% probability level. Associations between the serum calcium level, bone metabolism biomarkers, and calcium transporters were evaluated using Spearman correlation. SPSS 22 for Windows was used for statistical analysis and figure production. Data were expressed as mean and standard deviation.

## 3 RESULTS

### 3.1 Impact on body mass and dietary intake

The effects of diet modifications on body weight gain and feed efficiency ratio during the 8‐week intervention period are shown in Table 3. Compared with the standard diet, the feed efficiency ratio of the modified diets did not change significantly. The RS diet significantly reduced weight gain, whereas the DG, LA, and DGLA diets significantly increased weight gain.

**TABLE 3:** Body weight gain and dietary energy concentration of the rats during 8 weeks of intervention period.

Group	Body weight gain (%)				Dietary energy (kcal/day)			
	Mean ± SD	Median	Q1	Q3	Mean ± SD	Median	Q1	Q3
K	32.25 ± 6.03^b^	29.36	28.52	33.71	66.32 ± 9.11^a^	65.48	60.80	70.20
TP	36.13 ± 3.47^bc^	36.28	33.69	39.23	62.27 ± 8.10^b^	61.33	57.06	67.58
RS	22.91 ± 2.96^a^	22.76	21.62	24.20	54.38 ± 9.67^c^	54.51	48.59	59.85
DG	44.30 ± 9.18^cd^	46.20	37.47	51.08	78.52 ± 9.29^d^	78.28	73.53	84.97
LA	45.42 ± 4.11^d^	45.29	41.62	46.77	76.84 ± 8.84^d^	75.89	71.31	81.87
DGLA	42.87 ± 5.30^cd^	42.99	41.33	45.54	76.14 ± 8.25^d^	75.50	70.72	80.64

*Note*: Values are shown as mean and standard deviation (SD), median, the first quartile (Q1), and the third quartile (Q3). Tukey's test for ANOVA was used. ^a–d^ are mean values significantly different for *p* < .05.

### 3.2 Impact on the serum calcium levels

**FIGURE 2:**
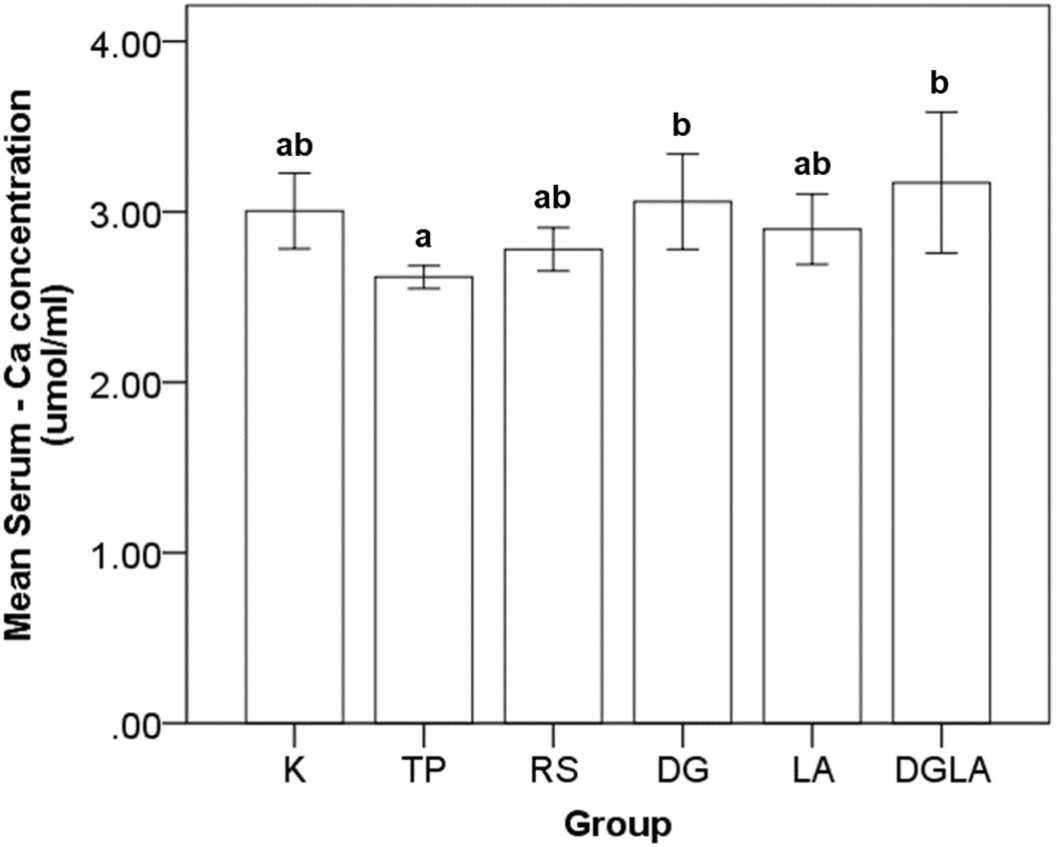
Calcium serum levels in different diet groups after 8‐week intervention period. Values are presented as mean and standard error. Tukey's test for ANOVA was used. ^a,b^are mean values significantly different for *p* < .05.

The serum calcium levels after the 8‐week intervention period are depicted in Figure 2. Compared with the control group, no significant differences were observed in the modified groups. However, significantly decreased serum calcium levels were observed in the TP group than in the DG and DGLA groups.

### 3.3 Impact on bone metabolism biomarkers

**FIGURE 3:**
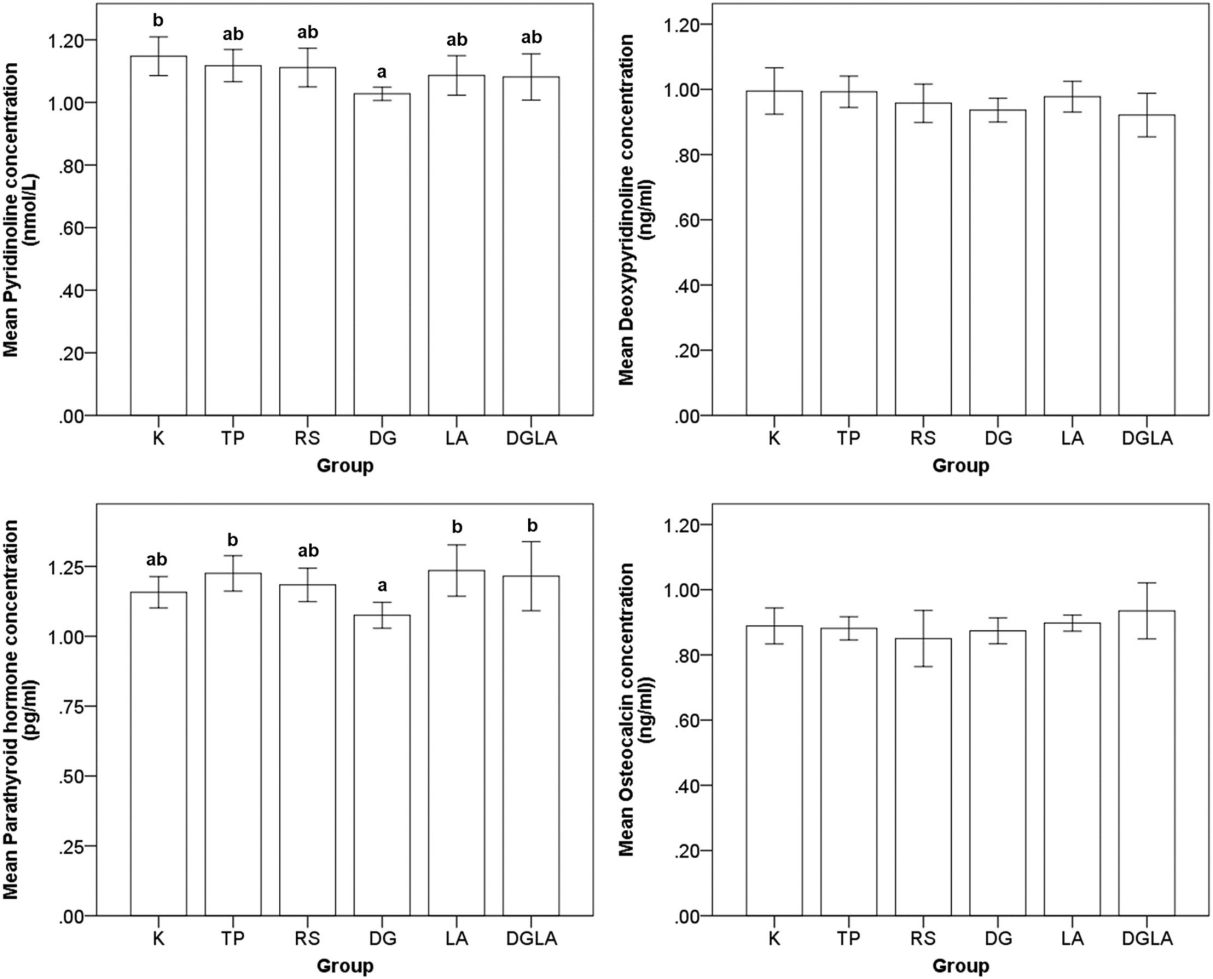
Pyridinoline, deoxypyridinoline, parathyroid hormone, and osteocalcin levels in serum after 8‐week intervention period. Values are presented as mean and standard error. Tukey's test for ANOVA was used. ^a,b^ are mean values significantly different for *p* < .05.

The effects of the modified diets on pyridinoline, deoxypyridinoline, parathyroid hormone, and osteocalcin are shown in Figure 3. Compared with the control group, only the DG group showed significantly decreased serum pyridinoline levels (by 10%), and the TP, LA, and DGLA groups showed significantly increased serum parathyroid hormone levels. Moreover, it has been observed that LA counteracted the effect of DG on parathyroid hormone in serum.

### 3.4 Impact on calcium transport in the duodenum and jejunum

**FIGURE 4:**
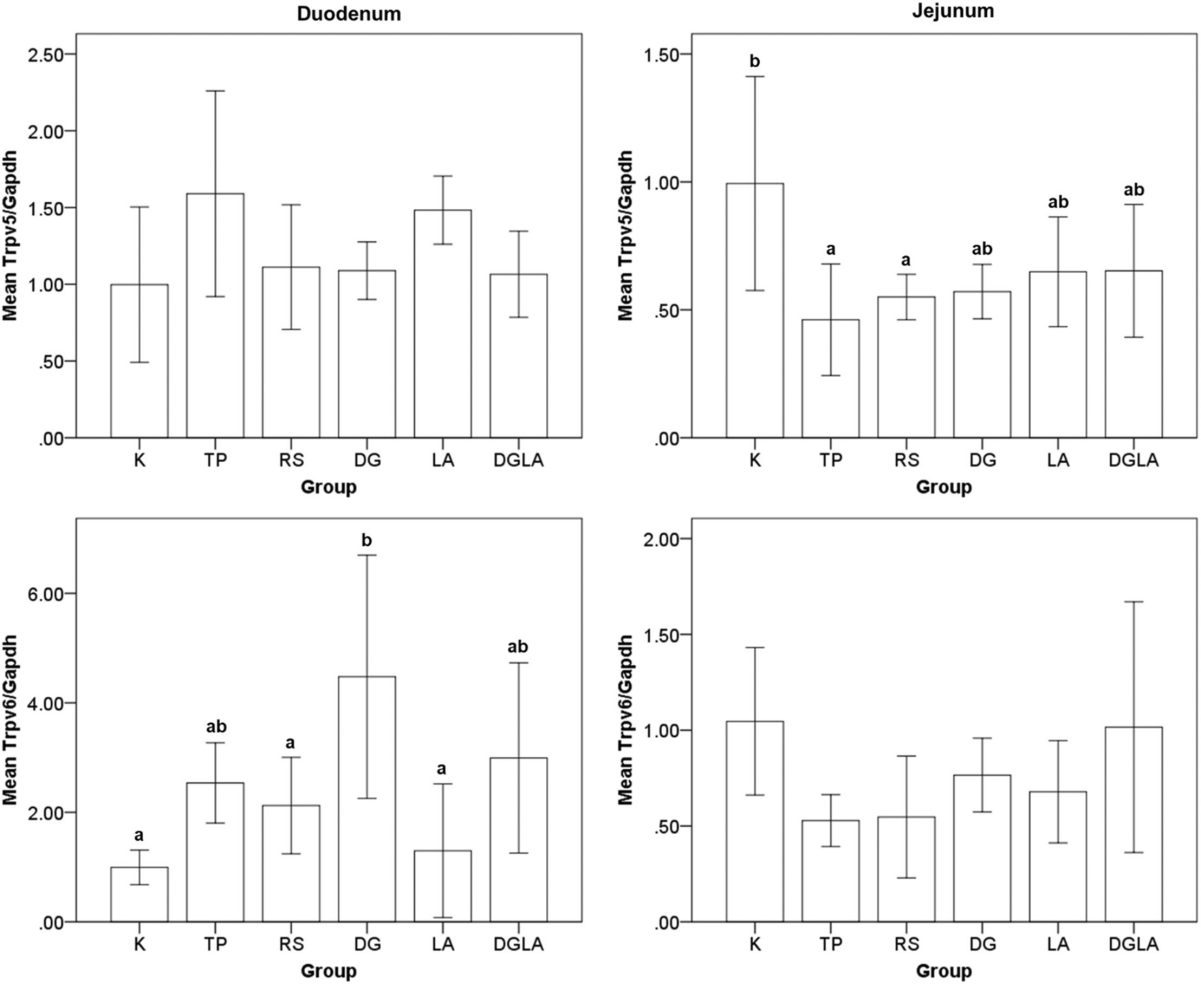
Trpv5 and Trpv6 mRNA expression in the duodenum and jejunum after 8‐weeks intervention period. Trpv5 and Trpv6 mRNA levels were measured by real‐time RT‐PCR and expressed relative to levels of Gapdh mRNA. Values (means ± SD) are expressed as arbitrary units. Tukey's test for ANOVA was used. ^a,b^ are mean values significantly different for *p* < .05.

The effects of the modified diets on calcium transport in the duodenum and jejunum are shown in Figure 4. After the 8‐week intervention period, the TP and RS groups showed significantly decreased Trpv5 levels in the jejunum by 54% and 45%, respectively, compared with the control. Meanwhile, the DG group showed 3.5‐fold increased Trpv6 levels in the duodenum.

### 3.5 Correlation between serum calcium levels, bone metabolism biomarkers, and calcium transporters

The correlation analysis presented in Table 4 was performed on the entire study group to assess the significant relationship between serum calcium levels, bone metabolism biomarkers, and calcium transport in the duodenum and jejunum. The serum calcium level was positively associated with the Trpv6 calcium transporter in the jejunum. Furthermore, the PYD level was positively correlated with the serum PTH and OC levels. Meanwhile, positive associations were also observed between the DPD level and the serum PTH and OC levels. The DPD level was positively correlated with the expression of Trpv5 in the duodenum.

**TABLE 4:** Spearman's correlation between calcium levels in organs and bone biomarkers in serum and histopathological bone structure.

Relationship	Coefficient	Significance^a^
Ca serum – Trpv6 jejunum	.309	.043
PYD – PTH	.401	.005
PYD – OC	.358	.012
DPD – PTH	.605	.000
DPD – OC	.586	.000
DPD – Trpv5 duodenum	.297	.042

*Abbreviations*: Ca, calcium; DPD, deoxypyridinoline; OC, osteocalcin; PTH, parathyroid hormone; PYD, pyridinoline; Trpv5, Transient receptor potential cation channel subfamily V member 5; Trpv6, Transient receptor potential cation channel subfamily V member 6.

^a^ A two‐tailed test of significance.

## 4 DISCUSSION

In our study, the intake of DG was found to have significant effects on calcium transport in the duodenum and pyridinoline serum concentration. These findings highlight the novel and important outcomes of our research. The improvement in calcium transport observed in the duodenum suggests that DG may enhance the absorption and utilization of calcium in the body. Additionally, the reduction in pyridinoline serum concentration indicates a potential role of DG in modulating bone remodeling processes and potentially reducing the risk of bone loss. These key results provide valuable insights into the impact of DG intake on calcium metabolism and bone health.

Previous studies have shown that isoflavones decreased bone resorption in postmenopausal women (Harkness et al., 2004; Ma et al., 2008; Yamori et al., 2002) and ovariectomized rats (Ishida et al., 1998; Ma et al., 2013; Tousen et al., 2016). The findings of the present study confirmed that this might indicate the preventive properties of DG in relation to bone density under healthy conditions. Previous research showing that isoflavones from soy can lower pyridoxine levels was verified in our work (Akhlaghi et al., 2020; Kanadys et al., 2021). In the present study, the reduction in parameters of bone resorption due to DG intake was associated with increased calcium transport in the duodenum and, consequently, an increased serum calcium level (but slightly). Previous studies reported that isoflavones might prevent BMD loss (Li et al., 2010) by increasing the intestinal absorption of calcium (Zafar et al., 2004). Isoflavones increase the intestinal absorption of calcium through an estrogen‐like mechanism as exogenous estrogen improves the intestinal absorption of calcium in ovariectomized rats (Breitman et al., 2003). Another study reported that DG interacts with serum calcium to selectively alter whole‐body BMD and BMC (Zafar et al., 2004). In addition, when serum calcium levels are low, isoflavones considerably decrease BMD and BMC, but when serum calcium levels are high, isoflavones enhance BMD and BMC (Nayeem et al., 2019). Serum albumin levels play a role in mediating the effects of DG on circulation calcium balance. Regulating effects of isoflavones on calcium status were also observed in premenopausal women supplemented simultaneously with calcium and soy isoflavones (Lu et al., 2018). Hence, the primary outcome of this study is that DG may increase BMD and mineral contents by enhancing calcium transport, reducing bone resorption, and modestly improving serum calcium levels.

Compared with the DG diet, RS and TP diets significantly decreased serum calcium levels, and this decrease was significantly supported by reduced calcium transport in the jejunum compared with the control group. This result may be attributable to the presence of phytic acid, oxalic acid, and fiber, all of which may reduce calcium availability (Janve & Singhal, 2018). In addition to nutritional components, RS contains tannins (Fen Zhang et al., 2011), phytic acid (Medic et al., 2014), fiber (Ferreira et al., 2015), saponin (Berhow et al., 2020), and oxalate (Xian et al., 2020). In particular, these antinutritional substances reduce the absorption of calcium and other minerals such as iron, copper, and zinc (Samtiya et al., 2020) by chelating them in the digestive system (Gibson et al., 2018).

This study also found that the inclusion of RS in the diet resulted in a significant reduction in weight gain. Soy protein, present in soybean, has been shown to improve insulin resistance and lipid levels by activating peroxisome‐proliferator‐activated receptors. These nuclear transcription factors regulate the expression of genes involved in glucose homeostasis, lipid metabolism, and fatty acid oxidation. Moreover, soy protein has the potential to reduce adiposity by modulating the expression of sterol regulatory element binding proteins (Velasquez & Bhathena, 2007).

Surprisingly, LA did not significantly influence calcium transport and bone metabolism biomarkers in this study. In contrast to the literature reports (Dai et al., 2020; Lee et al., 2019; Tang et al., 2006), the present study did not confirm that probiotics enhance calcium bioavailability and bone metabolism. Furthermore, our present study revealed that LA had a higher PTH concentration than DG. Thus, a synergistic effect between isoflavones and probiotics in bone metabolism was not confirmed in the present study. The results of the present study may be explained using healthy rats, and under healthy conditions without any bone or calcium disorders, this beneficial effect may not be observed (Harahap, Kuligowski, Schmidt, Kurzawa, et al., 2023; Harahap, Kuligowski, Schmidt, & Suliburska, 2023).

One potential explanation for the absence of a significant response and synergistic effect in the DGLA group could be the unknown interactions between active components, such as isoflavones and *L. acidophilus* that may have influenced the outcomes. The interplay between these components can result in a modulation of their individual effects, potentially leading to a diminished or negligible overall response. In agreement with previous research, the probiotics did not enhance the effects of isoflavones on lipid metabolism and the endocrine system in the animal model (Ali et al., 2004). Contrary to obtained results, evidence suggests an interaction between probiotics and daidzein/genistein. Mathey et al. conducted a study demonstrating the bone‐sparing effects of long‐term consumption of genistein and daidzein in ovariectomized rats. Moreover, the inclusion of *Lactobacillus casei* in the diet has shown a significant enhancement of isoflavones' protective effects on the skeletal system (Mathey et al., 2007). These findings underscore the potential benefits of combining specific dietary components and probiotics for promoting bone health but were not confirmed in this study.

The positive correlation between the analyzed parameters observed in this study partly confirmed that calcium homeostasis is regulated by bones and that bone‐forming and bone‐resorbing cells employ calcium signals to differentiate and activate (Blair et al., 2011). In the present study, calcium transport was related to serum calcium levels and bone resorption, and bone formation parameters were correlated with bone resorption to maintain optimal bone metabolism. Calcium release from internal storage and its input into the extracellular fluid regulate calcium signaling, which governs various cellular processes. Calcium signals in osteoclasts regulate gene transcription, differentiation, and bone resorption (Kajiya, 2012). Osteoclasts remove mineralized bones, and osteoblasts produce a bone matrix that becomes mineralized during bone remodeling. The remodeling cycle comprises three phases: resorption, in which osteoclasts eat old bones; reversal, in which mononuclear cells emerge on the bone surface; and formation, in which osteoblasts lay down new bones until the resorbed bone is completely replaced (Hadjidakis & Androulakis, 2006).

Overall, our study's focus on the impact of our intervention on calcium transport in the duodenum and jejunum provides valuable insights into the dynamics of body calcium turnover. The duodenum and jejunum play crucial roles in absorbing dietary calcium, which is essential for various physiological processes. Understanding the regulation of calcium transport in these regions contributes to our understanding of calcium homeostasis and its implications for overall health. Our findings contribute to the broader knowledge of body calcium turnover and can guide future research on optimizing calcium absorption and utilization.

### 4.1 Limitation of the study

The major strength of the present study is the intervention using probiotics and isoflavones, which has never been employed in the analysis of calcium transporters and bone metabolism biomarkers in healthy rats. In addition, the association between calcium transport and bone metabolism biomarkers in the regulation of calcium homeostasis was examined. These findings represent the originality of the study. However, this study is severely limited by analyzing only selected parameters of calcium transport, calcium status, and bone metabolism. In addition, this study did not examine vitamin D transport and vitamin D levels. In the context of bone regulation, calcitriol diffuses into cells and binds to complex vitamin D receptors. Through this mechanism, calcitriol exerts control over the absorption of calcium in the intestines, ensuring optimal uptake, and subsequently supplies an adequate amount of calcium to the bone matrix (Harahap, Kuligowski, et al., 2022; Harahap, Landrier, & Suliburska, 2022).

Moreover, another limitation of our study is the absence of fecal calcium analysis, which is essential for evaluating the potential inhibitory effects of phytic acid, dietary fiber, tannins, and saponins on calcium absorption, transport, and absorption. These components are known to interfere with the utilization of calcium in the gastrointestinal tract. Without measuring fecal calcium levels, we were unable to directly assess the impact of these inhibitory components on calcium excretion and absorption, which could have provided a more thorough understanding of the observed lack of effect of tempeh flour and soybean. Incorporating fecal calcium analysis in future studies would be valuable in elucidating the mechanisms underlying the relationship between these inhibitory components and calcium metabolism, and further investigating their role in the efficacy of isoflavones and probiotics. Addressing this limitation would strengthen the scientific rigor of future research in this area.

In light of our study's findings, there is a need for further research to explore the potential clinical implications and applications. The study's findings suggest potential future applications in osteoporosis therapy by exploring isoflavones and probiotics as complementary interventions, though further research is needed to understand their mechanisms and optimize treatment strategies. Future human studies should focus on investigating the long‐term effects of the intervention on clinical outcomes such as bone health, calcium metabolism, and related markers. Additionally, examining the optimal dosage, duration, and timing of the intervention, as well as evaluating its efficacy in specific populations (e.g., postmenopausal women and individuals with calcium‐related disorders), would be valuable for guiding future clinical practice and interventions.

## 5 CONCLUSION

The daidzein and genistein diet improves calcium transport in the duodenum and reduces pyridinoline serum concentration in healthy female rats. The tempeh and soybean diets reduce calcium transport in the jejunum. The combination of daidzein, genistein, and *L. acidophilus* diet does not synergistically affect calcium transport in the small intestine and bone metabolism in healthy rats. In order to further understand the role of daidzein and genistein in preventing bone resorption in osteoporosis, more research needs to be done.

## AUTHOR CONTRIBUTIONS

**Iskandar Azmy Harahap:** Conceptualization (lead); data curation (lead); formal analysis (lead); funding acquisition (lead); investigation (lead); methodology (lead); project administration (lead); resources (lead); software (lead); supervision (equal); validation (lead); visualization (lead); writing – original draft (lead); writing – review and editing (lead). **Maciej Kuligowski:** Methodology (equal). **Marcin Schmidt:** Methodology (equal). **Paweł A. Kołodziejski:** Methodology (equal). **Joanna Suliburska:** Conceptualization (lead); formal analysis (equal); investigation (equal); methodology (lead); project administration (lead); supervision (lead); writing – review and editing (equal).

## FUNDING INFORMATION

Publication was co‐financed within the framework of the Polish Ministry of Science and Higher Education program: “Regional Excellence Initiative” in the years 2019–2023 (No. 005/RID/2018/19)”, financing amount 12 000 000,00 PLN. In addition, this study secured the 2021 Young Scientist Research Grant from the Faculty of Food Science and Nutrition, Poznan University of Life Sciences, for Iskandar Azmy Harahap.

## CONFLICT OF INTEREST STATEMENT

The authors declare that they have no conflict of interest.

## ETHICS STATEMENT

The Local Ethics Committee for Experimental Animals has permitted the present study at the Poznan University of Life Sciences.

## CONSENT FOR PUBLICATION

The present paper, which is original, has not been published before and is not currently being considered for publication elsewhere.

## Data Availability

All data generated or analyzed during this study are available from the corresponding author on reasonable request.
